# VEGF Polymorphisms (*VEGF-936 C/T*, *VEGF-634 G/C* and *VEGF-2578 C/A*) and Cardiovascular Implications in Long COVID Patients

**DOI:** 10.3390/ijms25168667

**Published:** 2024-08-08

**Authors:** Angela Cozma, Adela Viviana Sitar-Tăuț, Olga Hilda Orășan, Violeta Briciu, Daniel Leucuța, Nicolae-Dan Sporiș, Andrada-Luciana Lazăr, Toma-Vlad Mălinescu, Andreea-Maria Ganea, Bianca Mihaela Sporiș, Călin Vasile Vlad, Mihaela Lupșe, Mădălina-Gabriela Țâru, Lucia Maria Procopciuc

**Affiliations:** 1 Department of Internal Medicine, “Iuliu Hațieganu” University of Medicine and Pharmacy, 400012 Cluj-Napoca, Romania; 2 Department of Infectious Diseases and Epidemiology, “Iuliu Hațieganu” University of Medicine and Pharmacy, 400348 Cluj-Napoca, Romania; 3 Department of Medical Informatics and Biostatistics, “Iuliu Haţieganu” University of Medicine and Pharmacy, 400349 Cluj-Napoca, Romania; 4 Department of Medical Oncology, Prof. Dr. I. Chiricuța Oncology Institute, 400015 Cluj-Napoca, Romania; 5 Department of Dermatology, “Iuliu Hațieganu” University of Medicine and Pharmacy, 400012 Cluj-Napoca, Romania; 6 Department of Cardiology, “Iuliu Hațieganu” University of Medicine and Pharmacy, 400012 Cluj-Napoca, Romania; 7 Department of Gastroenterology, Regional Institute of Gastroenterology “Prof. Dr. Octavian Fodor”, 400394 Cluj-Napoca, Romania; 8 Department of Medical Biochemistry, “Iuliu Haţieganu” University of Medicine and Pharmacy, 400012 Cluj-Napoca, Romania

**Keywords:** COVID-19, VEGF polymorphism, cardiovascular, pulse wave analysis, ejection fraction

## Abstract

The COVID-19 pandemic has raised awareness of the virus’s long-term non-pulmonary consequences. This study examined the relationship between genetic polymorphisms of VEGF and cardiac dysfunction and subclinical atherosclerosis in patients recovering from COVID-19. This study included 67 patients previously diagnosed with COVID-19. *VEGF-936C/T, VEGF-634G/C*, and *VEGF-2578C/A* statuses were determined. Conventional echocardiography and arterial parameters assessments were performed at inclusion and at six months after the first assessment. For *VEGF-936C/T*, dominant and over-dominant models showed a significant increase in ejection fraction at six months after COVID (*p* = 0.044 and 0.048) and was also a predictive independent factor for the augmentation index (β = 3.07; *p* = 0.024). The dominant model showed a rise in RV-RA gradient (3.702 mmHg) (*p* = 0.028 95% CI: 0.040–7.363), with the over-dominant model indicating a greater difference (4.254 mmHg) (*p* = 0.025 95% CI: 0.624–7.884). The findings for *VEGF-634G/C* were not statistically significant, except for a difference in TAPSE during initial evaluation, using the codominant model. For *VEGF-2578C/A*, a difference in ventricular filling pressure (E/E’ratio) was best described under the recessive model. Our research suggests that the *VEG-936C/T* genotype may impact the baseline level and subsequent changes in cardiac function and subclinical atherosclerosis. These findings offer valuable insights into the complex correlation between genetic polymorphisms and cardiovascular disfunction in long COVID patients.

## 1. Introduction

With the recent conclusion of the coronavirus disease 2019 (COVID-19) pandemic, there has been a growing focus on the non-pulmonary effects of the disease. In the context of the cardiovascular system, infection caused by severe acute respiratory syndrome-coronavirus 2 (SARS-CoV-2) might exhibit immediate symptoms and continue to persist during the recovery phase, potentially extending beyond this period [1]. Patients diagnosed with COVID-19 who possess pre-existing cardiovascular disease and associated risk factors, such as hypertension, diabetes, and obesity, exhibit inferior clinical outcomes [2]. Multiple studies have focused on identifying predisposing or exacerbating variables for COVID-19 and its effects on different organ systems. The primary factor leading to mortality in individuals with COVID-19 is hypoxic respiratory failure. This is a result of both parenchymal and vascular changes, which are comparable to the changes observed in acute respiratory distress syndrome (ARDS) caused by other factors [3], with microvascular dysfunction being the most probable cause [4]. In addition, it has been observed that individuals diagnosed with COVID-19 exhibit an overexpression of different proangiogenic factors, such as vascular endothelial growth factor (VEGF) [5]. High levels of oxidative stress developed during the active infection could promote pro-vasculogenic signaling as well as accelerate atherosclerosis [6]. The combination of these factors, along with the tendency for microthrombosis, may contribute to a higher risk of long-term damage to vital organs. This could potentially explain the symptoms experienced by patients with long-term COVID.

Vascular endothelial growth factor (VEGF) is an angiogenic factor produced by many cells, including macrophages, platelets, keratinocytes, and renal mesangial cells. The VEGF family consists of five proteins: VEGF-A, VEGF-B, placental growth factor (PIGF), VEGF-C, and VEGF-D. The first three are important regulators of blood vessel growth, while the last two modulate lymphangiogenesis [7]. Increased VEGF-A activity has been related to hypertension, inflammation, and cardiovascular diseases [8]. VEGF-A plays an important role in vasculogenesis and angiogenesis [9]. It is an intrinsic modulator with a crucial role in maintaining the integrity of the endothelial layer within the vascular wall [10]. The regulation of VEGF-A activity occurs when it binds to its receptors, VEGFR1 and VEGFR2 [11]. The human VEGF-A gene is located on chromosome 6p21.1 and has eight exons [12]. There are different genetic variants located in the VEGF-A gene that influence VEGF expression, influencing in fact the transcription of the gene and the VEGF activity [13]. The *VEGF-936C/T* polymorphism (*rs3025039*) is located in the 3′ untranslated region of the VEGF-A gene and is associated with lower protein production. This variant is linked to cardiovascular diseases [14]. The *VEGF-936 C/T* genetic variant has been implicated in the pathogenesis of thrombosis, as evidenced by its association with arteriovenous fistula thrombosis [9]. The *VEGF-634G/C* polymorphism (*rs2010963*) is a point mutation in the 5′UTR region of the VEGF-A gene. This genetic polymorphism influences VEGF-A expression and VEGF levels, influencing cholesterol, HDL levels, and blood pressure [15]. The *VEGF-2758C/A (rs699947)* polymorphism is located in the promoter region of VEGF-A, which influences total cholesterol and HDL levels and is associated with an increased risk for cardiovascular diseases [15]. The *VEGF-2578C/A* genetic variant also exhibited a heightened susceptibility to peripheral artery disease and diabetic retinopathy [16].

Although there are not many data regarding COVID-19, earlier research has shown that VEGF’s ability to promote vascular permeability plays a significant role in the pathophysiology of acute lung injury and (ARDS). Moreover, VEGF is recognized as an indirect procoagulant that modifies endothelial cell hemostatic properties [17].

## 2. Objectives

The main aim of this study is to examine the possible correlation between VEGF polymorphisms and cardiac dysfunction and subclinical atherosclerosis in patients recovering from COVID-19. Comprehending the hereditary elements that could make individuals more susceptible to these issues is essential for assessing risk, intervening early, and providing tailored medical treatment.

## 3. Results

### 3.1. Patient Characteristics and Demographics

The studied group (n = 67) comprised newly diagnosed COVID-19 patients in the age range of 19–75 years (mean age 41.26 ± 11.72 years), with a higher frequency of females (67.16%). The baseline demographic and clinical characteristics are shown in Table 1.

For the genotyped SNPs, only *VEGF-936 C/T* was in Hardy–Weinberg equilibrium. The genotype and allele frequencies of *VEGF-936 C/T, VEGF-634 G/C*, and *VEGF-2758 C/A* polymorphisms in the selected patients are detailed in Table 2.

### 3.2. Effect of VEGF-936 C/T on Cardiovascular Dysfunction and Subclinical Atherosclerosis

We investigated the association between *VEGF-936 C/T* and subclinical atherosclerosis, evaluated by IMT (intima–media thickness) during the six months after the initial evaluation. The data were analyzed using a variety of genetic models, including codominant, dominant, recessive, over-dominant, and log-additive. The results are shown in Table 3, which suggests that the *VEGF-936 C/T* polymorphism is linked to differences in IMT reduction, with the T allele carriers consistently showing greater reductions over six months than the C/C homozygotes, who were used as a reference.

A potential fit for the recessive and log-additive models was observed in the case of brachial AIx (augmentation index) increases over 6 months from the first examination. Under the recessive model (*p* = 0.052), individuals with the C/C-C/T genotype (n = 29) had a mean AIx difference of 3.569 ± 2.920%, while those with the T/T genotype (n = 3) exhibited a markedly higher mean AIx difference of 32.633 ± 40.771% (95% CI: 0.854–57.270). Under the log-additive model (*p* = 0.057), a mean AIx difference of 12.579 (95% CI: 0.101–25.060) was observed. These findings suggest a potential association between an increasing allele count and an increase in brachial AIx, although further investigation is warranted to confirm these trends. Variations in AIx were observed under the log-additive model at the time of the initial evaluation, with a mean decrease of 4.412% for each additional allele, although not statistically significant (*p* = 0.056).

We built a multiple linear regression model predicting AIx Ao as a function of *VEGF-936 C/T* log-additive model and adjusted for age, BMI, sex, SBPao (aortic central systolic blood pressure), and smoking status (Table 4).

The *VEGF-936C/T* polymorphism significantly influences the aortic AIx at baseline and was the only parameter regarding arterial function that had statistical significance at this early time point (for PWVao, the multivariate analysis did not reveal a significant association). *VEGF-936C/T* polymorphism is associated with a lower AIx, suggesting a potential protective effect against endothelial dysfunction. However, this effect does not persist at 6 months, indicating that other factors may become more relevant over time.

The dominant and over-dominant models (*p* = 0.044 and 0.048, respectively) demonstrated significant variations in EF over a period of six months following the initial evaluation. Using the dominant model, individuals carrying the C/T-T/T genotype showed an average enhancement in EF of 1.50%, whereas the comparison group with the C/C genotype displayed an average decline in EF of 2.25%. The mean disparity between these two groups amounted to 3.75% (95% CI: 0.276–7.224). According to the over-dominant model, individuals with the C/T genotype had a mean increase of 1.80% in EF over a six-month period, while the reference group (C/C-T/T) experienced a mean drop of 0.692% in EF during the same timeframe. The observed mean difference in EF variation between the two groups was 3.80% (95% CI: 0.200–7.399). Furthermore, all the employed models, with the exception of the recessive one, demonstrated statistical significance when used to analyze EF six months after the first evaluation (Table 5). These outcomes indicate that the *VEGF-936 C/T* genetic variant may have an impact on both the initial level of EF and its subsequent changes in individuals affected by viral infections.

Furthermore, we built a multiple linear regression model predicting EF as a function of the *VEGF-936 C/T* dominant model (C/T-T/T vs. C/C) and adjusted for SBPAo at 6 months + BMI (kg/m2) + GLS at 6 months + E/A at 6 months, and *VEGF-936* remained statistically significant (coefficient of 4.6 (95% CI 1.72–7.49), *p* = 0.003) (Table 6).

Variations in TAPSE were observed under the log-additive model six months after the initial evaluation, with a mean decrease of 3.57 mm for each additional allele, although the significance threshold was not met (*p* = 0.050). A significant association was observed under the codominant and over-dominant models for the RV-RA gradient increase measured at one month after COVID diagnosis (*p* = 0.028 and 0.025, 95% CI: 0.040–7.364 and 0.624–7.884), The mean increase in the RV-RA gradient, compared to the reference group, was found to be 3.702 mmHg and 4.254 mmHg for the codominant and over-dominant models, respectively (*p* = 0.028, 95% CI: 0.040–7.363, and *p* = 0.025, 95% CI: 0.624–7.884). The recessive model revealed a potentially significant increase in the mitral E/A ratio in the T/T group (mean difference 0.372, *p* = 0.046, 95% CI: 0.015–0.728).

In multivariate analysis, the *VEGF-936C/T* polymorphism was found to be an independent predictive factor for EF at 6 months. However, at baseline, none of the predictive factors, including the *VEGF-936C/T* genotype, significantly influenced EF. This indicates that the genetic influence of the *VEGF-936C/T* polymorphism may become more apparent over time.

### 3.3. Effect of VEGF-634 G/C on TAPSE (Right Ventricular Dysfunction)

Employing the codominant model, a significant difference regarding TAPSE determined at the first evaluation was observed between the patients exhibiting the C/G genotype (n = 4) compared with the G/G genotype (n = 31), with a mean difference of 3.184 mm in favor of the G/G group (*p* = 0.046, 95% CI: 0.164–6.204).

### 3.4. Effect of VEGF-2578 C/A on Diastolic Function

The effect of *VEGF-2578 C/A* on cardiac relaxation patterns was not significant under any of the employed genetic polymorphisms. Nevertheless, filling pressure patterns varied significantly over a period of six months when the codominant, over-dominant, and recessive models were analyzed. The best fit was for the recessive model, with a mean difference between the C/C group (n = 6, with an overall decrease in E/E’ ratio of 1.346) and the A/A-A/C group (n = 21, with an overall increase in E/E’ ratio of 0.862) of 2.209 (*p* = 0.013, 95% CI: 0.578–3.439).

### 3.5. Haplotypic Analysis

In the present investigation, we conducted a haplotypic analysis of the three VEGF polymorphisms: *VEGF-936C/T, VEGF-634G/C*, and *VEGF-2578C/A* (Table 7). Our findings indicate that there is no statistically significant association between these polymorphisms and several echocardiographic parameters, including GLS, E/A ratio, and EF. Additionally, no significant relationship was observed between these VEGF polymorphisms and arterial stiffness parameters, specifically brachial Aix. However, marginally non-significant associations were observed for the CGC haplotype regarding the PWV/GLS ratio variation during the observation period (*p* = 0.092), the CGC haplotype with TAPSE at six months (*p* = 0.101), and the TGC haplotype with IMT variation during the observation period (*p* = 0.055). This finding implies that, within the confines of our investigation, these genetic differences may have a limited impact on the cardiovascular parameters that were evaluated. Furthermore, we did not find any statistically significant associations between haplotypes and the severity of COVID-19 with either of the dominant, recessive, or additive models.

## 4. Discussion

Vascular endothelial growth factor is an angiogenic factor essential for endothelial cell growth [18], whose expression is induced by hypoxia [19]. Its functional importance from the embryogenesis period has been underlined in studies on knockout mice. In this situation, heterozygotes were not viable for more than 11–12 days because of abnormalities in the development of the vascular system [20]. Additionally, VEGF-A not only regulates vascular permeability [21] but also modulates the expression of intercellular adhesion molecule-1 and vascular cell adhesion molecule-1 [22].

Regarding the implication of VEGF polymorphisms in cardiovascular diseases and endothelial dysfunction, the results are contradictory. Amoli et al. investigated VEGF gene mRNA expression in subjects with abnormalities of the coronary arteries and found a decreased expression of the vascular endothelial growth factor gene in human peripheral blood mononuclear cells in patients with significant stenosis of the coronary arteries [23]. Furthermore, Cui et al. showed the association of two VEGF SNPs (*rs699947* and *rs2010963*) with coronary artery stenosis in a study conducted on a Chinese population with coronary artery disease that had undergone coronary angiography [24]. In a recent meta-analysis, Ma et al. found an association between the *rs3025039, rs699947*, and *rs2010963* VEGF polymorphisms and predisposition to coronary artery disease [25]. VEGF levels increase in the acute phase of myocardial infarction (MI) [26]. The cause of this may be the hypoxic state induced by myocardial ischemia. The acute phase proteins (C reactive protein, amyloid A protein) present the highest serum values on day three in MI patients and correlate with the peak of VEGF levels on day 7 [27]. The correlation between elevated VEGF serum levels and inflammation is supported by the results of a study conducted by Eržen et al. Forty-one patients with MI in the stable phase and 25 controls were enrolled in their study. The results showed significantly increased serum values of the inflammation markers IL-6 and CRP in patients. Also, they found a correlation between VEGF and IL-6 and IL-8 levels, with IL-6 being an independent determinant of VEGF. No relationship was found between VEGF serum levels and atherosclerosis [28].

It is important to mention that in our study, the VEGF polymorphism *rs2010963* was associated with variations in IMT and EF. Additionally, Kangas-Kontio et al. evaluated the same three polymorphisms we investigated in our study in a cohort of survivors of acute myocardial infarction. Men who had the CT genotype (*VEGF-936 C/T*) demonstrated elevated values of IMT in comparison to those with the CC genotype. When considering all three polymorphisms, it was observed that males with the CCT haplotype and women with the CCC haplotype exhibited a significantly larger intima–media thickness (IMT) [29].

IMT is a valuable atherosclerosis marker used worldwide and an established predictor for cardiovascular events [30]. A major role in the development of atherosclerosis is endothelial dysfunction through dysregulated homeostasis of nitric oxide synthesis or activity [31]. The impact of VEGF polymorphisms on VEGF serum levels and endothelial function was established in a study conducted by Steffensen et al. [32].

In this study, we did not find any association between the three tested polymorphisms of VEGF and COVID-19 severity. However, in other studies, VEGF-A presented increased levels in SARS-CoV-2 patients and was also associated with disease severity [33].

The serum values of VEGF proved to be an important marker in monitoring and predicting the evolution of the disease. In the case of patients with COVID-19, higher serum levels were associated with a more severe course of the disease [34]. Consequently, VEGF is considered a possible therapeutic target, especially in the case of patients suffering from severe forms of the disease, such as ARDS [35].

Endothelial dysfunction plays a major role in the pathogenesis of COVID-19 [36,37]. There is evidence that endothelial dysfunction, through mechanisms such as oxidative stress, disturbance of nitric oxide homeostasis, endothelial insult, inflammation, hypercoagulability, and adhesion disorders, triggers a series of undesirable events with an impact on the prognosis of patients suffering from COVID-19 [36,38]. Additionally, the presence of the SARS-Cov-2 virus was demonstrated in the endothelial cells of the capillaries in several organs [39,40] and it might play a role in the endothelial dysfunction associated with COVID-19. Moreover, the cardiac insult associated with SARS-Cov-2 infection could also be induced by the direct infection of the myocardium by the virus, as SARS-COV-2 antigens were found in autopsies of patients diagnosed with COVID-19 [41,42].

Our multiple regression models revealed that SBPAo is an independent predictive factor for AIx Ao, a finding consistent with previous studies and further validating our results [43]. The lack of association between SBPAo and EF in our model could be attributed to the characteristics of selected patients (without prior hypertension) and also due to the relatively short monitoring period. It is important to note that the earliest sign of cardiac dysfunction in hypertensive patients typically manifests as alterations in diastolic function, rather than in systolic function [44].

## 5. Materials and Methods

### 5.1. Study Design

This study incorporated a cohort of 67 patients who had previously been infected with SARS-CoV-2 and had received a COVID-19 diagnosis in the Clinical Hospital of Infectious Diseases, Cluj-Napoca. Inclusion criteria for this study were ages between 18 and 75 years and a positive SARS-CoV-2 diagnosis within the past one month at the time of their first evaluation. Exclusion criteria involved the presence of prior cardiovascular conditions, including hypertension, ischemic heart disease, documented arrhythmias (past or present), or diabetes mellitus. Patient stratification was based on the severity of the disease [45]. Every individual involved in this study underwent a cardiovascular evaluation at two separate moments in time. The initial assessment was conducted within a time frame of up to one month after their diagnosis of SARS-CoV-2. The second evaluation was carried out after a period of six months following the initial assessment. During these evaluations, a comprehensive assessment of cardiac and arterial parameters was conducted using standard echocardiography techniques, global longitudinal strain, intima–media thickness (IMT) as well as pulse wave analysis (PWA). We mention that all investigations (IMT measurement, PWA, echocardiographic determinations, and GLS measurement through speckle tracking) were conducted in accordance with the guidelines set forth by the European College of Cardiology. The collection of demographic data, as well as the patients’ previous medical records and their subjective accounts of symptoms and overall health throughout the SARS-CoV-2 infection, was conducted in a methodical manner, utilizing a pre-established questionnaire. All investigations were conducted at University Hospital C.F., Cluj-Napoca.

### 5.2. Sample Collection

During the initial presentation, blood samples were collected to conduct a complete blood count (CBC). Additionally, the levels of NT-proBNP and troponin I were determined to assess the presence of any potential subclinical cardiac injury that may have occurred as a result of the SARS-CoV-2 infection. The blood samples utilized for the determination of VEGF status were appropriately stored within a temperature range of 2–8 °C until processing.

### 5.3. Clinical Assessment

Arterial blood pressure was measured in supine position following a five-minute period of rest alongside other parameters for pulse wave pressure, Aortic Pulse Pressure (PPao), Aortic Augmentation Index (AAix), Pulse Wave Velocity of the Aorta (PWVao), using an arteriograph (TensioMed, Budapest, Hungary). Global longitudinal strain of the left ventricle (LvGLS), other basic echocardiographic parameters, and intima–media thickness (IMT) were determined using a Vivid™ T8, GE HealthCare. All the parameters were determined at both evaluations.

### 5.4. Genotyping

DNA isolation: 2 mL of blood was drawn in tubes containing ethylene diamine tetra acetic acid as an anticoagulant. Genomic DNA was extracted using a Zymoreasearch kit (Quick-DNAMiniprep, Kit-Zymo Research Corporation, Freiburg, Germany). All the DNA samples were stored at −20 °C until use for PCR analysis.

PCR amplification: *VEGF-936C/T (rs3025039), VEGF-634G/C (rs2010963)*, and *VEGF-2578C/A (rs699947)* SNPs genotyping was performed using the PCR–RFLP methods described by Papazoglou et al. (2004) and Liu et al. (2009) and optimized in our laboratory [46,47,48]. We used specific forward and reverse primers for each genetic variation, as presented in Table 8. The amplification occurred in 25 μL mixture in an iCycler C1000 BioRad (Bio-Rad Life Science, Hercules, CA, USA) using the following reagents: 20 ng genomic DNA, 0.2 μM primers, 200 mM dNTPs (dATP, dGTP, dCTP, dTTP), 2.0 mM MgCl 2, 0.625 U Taq polymerase in specific buffer (Taq buffer, 20 mM Tris-HCl (pH 8.0), 1 mM DTT, 0.1 mM EDTA, 100 mM KCl, 0.5% (*v*/*v*) Nonidet P40, 0.5% (*v*/*v*) Tween20 and 50% (*v*/*v*) glycerol). The PCR conditions were as follows: initial denaturation at 95 °C for 60 s, 34 cycles of denaturation at 95 °C for 10 s, primer annealing at 68.2 °C for 30 s (*VEGF-936C/T*), 53.7 °C for 20 s (*VEGF-634G/C* polymorphism), and 67.2 °C for 30 s (*VEGF-2578C/A*), primer extension at 72 °C for 20 s, and a final extension for 30 s at 72 °C. The specificity of the PCR amplification was verified by migration on 2% agarose gel stained with 10 mg/mL ethidium bromide solution. The primers were from Eurogentec (Kaneka Eurogentec S.A. Biologics Division, Sering, Belgium).

**Figure 1:**
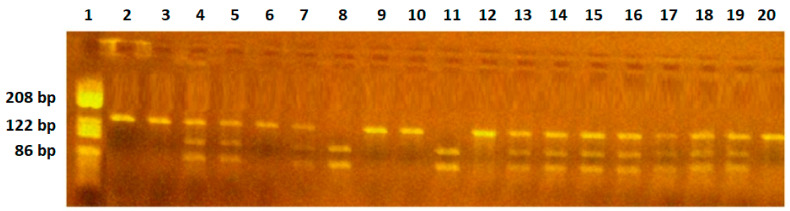
*VEGF-936C/T* polymorphism identification. Lane 1—pBRHaeIIIDigest DNA molecular marker; lanes 2, 3,6,9,10,12,20—CC genotype, fragment of 208 bp; lanes 4,5,7,13,14,15,16,17,18,19—CT genotype, fragments of 288 bp, 122 bp, 86 bp; lane 8,11—TT genotype, fragments of 122 bp and 86 bp.

**Figure 2:**
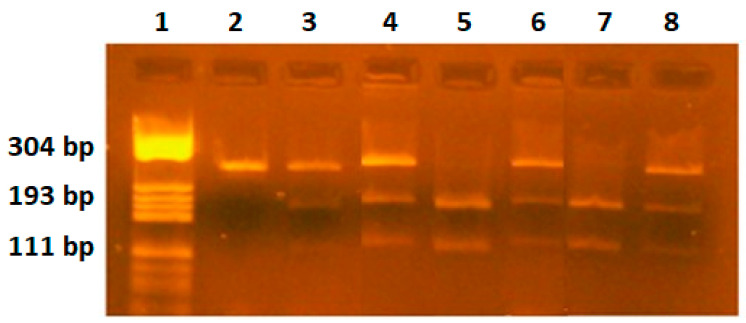
*VEGF-634G/C* polymorphism identification. Lane 1—pBRHaeIIIDigest DNA molecular marker; lanes 2—PCR product, fragment of 304 bp; lanes 3,4,6,8—GC genotype, fragments of 304 bp, 193 bp, 111 bp; lanes 5,7—GG genotype, fragments of 193 bp and 111 bp.

**Figure 3:**
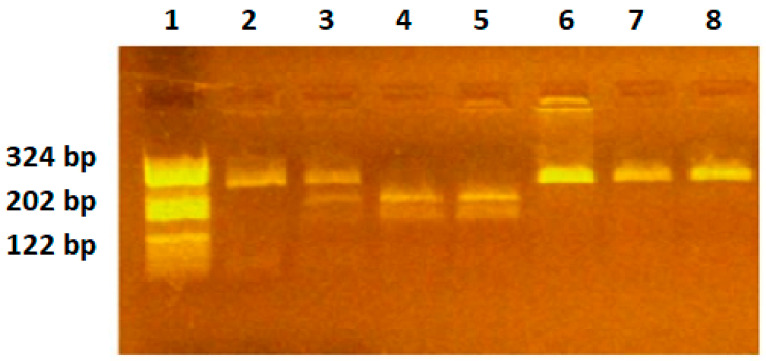
*VEGF-2578C/A* polymorphism identification. Lane 1—pBRHaeIIIDigest DNA molecular marker; lanes 2,6,7,8—CC genotype, fragment of 324 bp; lanes 3—CA genotype, fragments of 324 bp, 202 bp, 122 bp; lanes 4,5—AA genotype, fragments of 202 bp and 122 bp.

RFLP analysis: 6 μL PCR products was digested for 3 h with 2U-specific endonucleases, NlaIII (*VEGF-936C/T*), BsmFI (*VEGF-634G/C*), BglII (*VEGF-2578C/A*). The digested products were separated by migration in 3% agarose gel stained with 10 mg/mL ethidium bromide solution. Gels were visualized on a UV transilluminator. The C936 allele yields an undigested fragment of 208 bp, while the T936 allele yields two fragments of 122 bp and 86 bp. The G634 allele yields two fragments of 193 bp and 111 bp, while the C634 allele yields an undigested fragment of 304 bp. The C2578 allele yields an undigested fragment of 324 bp, while the A2578 allele yields two fragments of 202 bp and 122 bp (Figure 1, Figure 2 and Figure 3). The restriction enzymes were from New England Biolabs (New England Biolabs UK, Ltd., Hitchin, UK).

### 5.5. Statistical Analysis

Descriptive statistics in the form of counts and percentages were used to present categorical data, and mean and standard deviation were used to present quantitative data. The Hardy–Weinberg equilibrium exact test and the associations between SNPs and quantitative and qualitative echocardiographic parameters were computed using the package SNPassoc 2.1-0 [49]. For these associations, we assessed the following models: codominant, dominant, recessive, over-dominant, and log-additive. Haplotype frequencies were determined for both the severe and control groups. To evaluate the relationship between HLA haplotypes and the disease’s severity or quantitative echocardiographic parameters, score tests were employed [50]. The analysis of all haplotypes was conducted using version 1.9.3 of the haplo.stats R package [51]. In all statistical tests conducted, a two-tailed *p*-value and a significance threshold of 0.05 were employed. The statistical computations were performed using the R environment for statistical computing and graphics (R Foundation for Statistical Computing, Vienna, Austria), version 4.3.1 [52].

## 6. Conclusions

*VEGF-936 C/T* polymorphism: the T allele is associated with a continuous decrease in IMT over six months compared to homozygous C allele carriers. A potential correlation exists between a higher number of alleles and an increase in brachial AIx, indicating the need for further investigation.

Significant variations were identified in indices like EF, TAPSE, RV-RA gradient, and mitral E/A, suggesting *VEGF-936 C/T*’s influence on cardiac function in COVID-19 infected patients.

The *VEGF-936C/T* polymorphism is an independent predictor of left ventricular dysfunction (assessed by ejection fraction) and endothelial dysfunction (measured by augmentation index), but it is not a predictor of arterial stiffness (assessed by pulse wave velocity studies).

No notable associations were found between VEGF polymorphisms and the severity of COVID-19.

## 7. Study Limitations

The small number of patients enrolled, the single-center experience, and the lack of a control group are the two main limitations of the present study. It is important to mention that a proper control group would have been especially difficult to assemble, taking into consideration that the actual study started enrolling patients almost two years after the beginning of the pandemic. Healthy patients with no previous contact with the SARS-CoV-2 are especially hard to identify and recruit. Studies based on a larger cohort at additional sites are needed to verify our findings.

## Figures and Tables

**Table 1:** Demographics and clinical characteristics.

Characteristics	Categories/Types	N (%)
Sex	Male	45 (67.16)
	Female	22 (32.84)
Ethnicity	Romanian	62 (92.53)
	Hungarian	5 (7.47)
Smoking habit	Yes	18 (26.87)
	No	49 (73.13)
BMI (kg/m^2^)	Underweight	3 (4.47)
	Normal	39 (58.20)
	Overweight	17 (25.37)
	Obese	8 (11.94)
	Mean	24.45 ± 4.64
Comorbidities	History of neoplasia	2 (2.98)
	Autoimmune disease	9 (13.43)
	Liver disease	6 (8.95)
	Renal disease	2 (2.98)
	Pulmonary disease	8 (11.94)

**Table 2:** Genotype and allele frequency.

***VEGF-936*** ***C/T*** ***(rs3025039)***	**Genotype**	**Count (Proportion)**
	C/C	42 (0.63)
	C/T	19 (0.28)
	T/T	6 (0.09)
	Alleles	Count (Proportion)
	C	103 (0.77)
	T	31 (0.23)
***VEGF-634*** ***C/G*** ***(rs2010963)***	Genotype	Count (Proportion)
	G/G	58 (0.87)
	G/C	9 (0.13)
	C/C	0 (0)
	Alleles	Count (Proportion)
	G	125 (0.93)
	C	9 (0.07)
***VEGF-2578*** ***C/A*** ***(* *rs699947* *)***	Genotype	Count (Proportion)
	C/C	14 (0.21)
	C/A	23 (0.34)
	A/A	30 (0.45)
	Alleles	Count (Proportion)
	C	51 (0.38)
	A	83 (0.62)

**Table 3:** *VEGF-936 C/T* impact on IMT six months after initial evaluation.

Model	N	Mean (mm) ± SD	Mean Difference	95% CI	*p*
Co-dominant					
C/C	17	0.045 ± 0.021	Reference		0.017
C/T	10	0.010 ± 0.0281	0.055	0.011–0.121	
T/T	3	0.110 ± 0.023	0.155	0.023–0.155	
Dominant					
C/C	17	0.045 ± 0.021	Reference		0.022
C/T-T/T	13	0.033 ± 0.025	0.014	0.014–0.142	
Recessive					
C/C-C-T	27	0.024 ± 0.017	Reference		0.017
T/T	3	0.110 ± 0.023	0.134	0.030–0.239	
Over-dominant					
C/C-T/T	20	0.022 ± 0.022	Reference		0.395
C/T	10	0.010 ± 0.028	0.032	0.040–0.104	
Log-additive					
0, 1, 2			0.069	0.025–0.114	0.004

**Table 4:** Multiple linear regression analysis of predictors for aortic AIx.

	At Baseline			At 6 Months		
	β	95% CI	*p*	β	95% CI	*p*
*VEGF-936C/T* log additive	−3.07	−5.65–0.48	0.024	−1.85	−5.4–1.7	0.318
Age (years)	0.48	0.24–0.72	<0.001	0.52	0.27–0.78	<0.001
BMI (kg/m^2^)	−0.46	−0.89–−0.02	0.044	−0.79	−1.26–−0.31	0.003
Sex (Male vs. Female)	−11.46	−15.2–−7.73	<0.001	−10.93	−16.7–−5.16	0.001
SBPao (mmHg)	0.32	0.15–0.5	<0.001	0.32	(0.06–0.58)	0.026
Smoking (Yes vs. No)	4.18	−0.17–8.53	0.064	1.06	−4.76–6.88	0.724

**Table 5:** *VEGF-936 C/T* impact on EF at six months after initial evaluation.

Model	N	Mean (%) ± SD	Mean Difference	95% CI	*p*
Co-dominant					
C/C	16	54.88 ± 0.90	Reference		0.008
C/T	11	59.91 ± 1.29	5.034	2.089–7.979	
T/T	2	58.00	3.125	−2.514–8.764	
Dominant					
C/C	16	54.88 ± 0.90	Reference		0.002
C/T-T/T	13	59.62 ± 1.10	4.740	0.014–0.142	
Recessive					
C/C-C-T	27	56.93 ± 0.88	Reference		0.747
T/T	2	58.00	1.074	−5.3959–7.544	
Over-dominant					
C/C-T/T	18	55.22 ± 0.83	Reference		0.003
C/T	11	59.91 ± 1.29	4.687	1.7997–7.574	
Log-additive					
0, 1, 2			3.202	0.856–5.548	0.012

**Table 6:** Multiple linear regression analysis of predictors for ejection fraction.

	At Baseline			At 6 Months		
	β	95% CI	*p*	β	95% CI	*p*
*VEGF-936C/T* dominant (CT-TT vs. CC)	−1.15	−5.53–2.23	0.493	4.6	1.72–7.49	0.003
SBPao (mmHg)	−0.32	−0.72–0.08	0.111	0	−0.13–0.13	0.985
BMI (kg/m^2^)	−0.02	−0.16–0.13	0.818	−0.31	−0.64–0.02	0.060
GLS (%)	−0.09	−0.88–0.69	0.811	0.19	−0.36–0.74	0.484
E/A	2.42	−2.50–7.34	0.325	−3.48	−9.83–2.87	0.267

**Table 7:** The most frequent haplotypes for the entire sample.

VEGF-936C/T	VEGF-634G/C	VEGF-2578CA	Probability
C	G	A	0.428
C	G	C	0.274
T	G	A	0.142
T	G	C	0.089
C	C	A	0.050
C	C	C	0.018
T	C	A	<0.001

**Table 8:** Primers used for polymorphism detection.

Genetic Variations	Primers	PCR Fragment	Restriction Enzyme	RFLP Fragments
*VEGF-936C/T*	F: 5′-AAGGAAGAGGAGACTCTGCGCAGAGC-3′ R:5′-TAAATGTATGTATGTGGGTGGGTGTGTCTACAG-3′	208 bp	*NlaIII*	*C936* allele: 208 bp *T936* allele: 122 bp, 86 bp
*VEGF-634G/C*	F: 5′-ATTTATTTTTGCTTGCCATT-3′ R: 5′-GTCTGTCTGTCTGTCCGTCA-3′	304 bp	*BsmFI*	*G634* allele: 193, 111 bp *C634* allele: 304 pb
*VEGF-2578C/A*	F: 5′-GGATGGGGCTGACT AGGTAAGC-3′ R: 5′-AGCCCCCTTTTCCT CCAAC-3′	324 pb	*BglII*	*C2758* allele: 324 bp *A2758* allele:202 bp, 122 bp

F—Forward; R—reverse.

## Data Availability

The data that support the findings of this study are available on request from the corresponding author. The data are not publicly available due to privacy or ethical restrictions.
